# Supplementary material for: Automated Path Searching Reveals the Mechanism of Hydrolysis Enhancement by T4 Lysozyme Mutants
Source: Int J Mol Sci. 2022 Nov 23;23(23):14628. doi: 10.3390/ijms232314628 (PMC9736071; doi:10.3390/ijms232314628)
Supplement: Supplementary file 1 [file ijms-23-14628-s001.zip › ijms-1981596-supplementary.pdf]

Article: Supplementary materials

Automated Path Searching Reveals the Mechanism of Hydrolysis Enhancement by T4 Lysozyme Mutants

Kun Xi and Lizhe Zhu *

Warshel Institute for Computational Biology, School of Life and Health Sciences, School of Medicine, The Chinese University of Hong Kong, Shenzhen, Shenzhen 518172, China
* Correspondence: zhulizhe@cuhk.edu.cn

Methods

Generate Initial Structures of the Three T4L variants

By using the crystal structures of Ground (G) State (PDB ID:3DMV) [1] and Excited (E) State (PDB ID: 2LCB) [2] as the templates, the initial structures of the G and E states for other two T4L variants (L99A,G113A and L99A,G113A,R119P) were generated by homology modelling via Modeller [3] (the E state of T4L-L99A,G113A,R119P, PDB ID:2LC9 [1]).

TAPS Optimization

The initial path of the G/E interconversion for the three T4L variants were generated by targeted molecular dynamics (MD) [4], see Table S1 for the parameter set. Then, the initial path was optimized by our recently-developed path searching method—the travelling-salesman based automated path searching method (TAPS) [5,6], see Table S2 for the details of the parameter set. The flowchart of the recently-matured TAPS method is shown in Figure S1.

Free Energy Landscape Calculation

After the path optimization for the three T4L variants (convergence check by performing the calculation of multidimensional scaling (MDS) [7] and $\sqrt{\langle z \rangle}$ [8–10] as shown in Figure S2), we chose the final converged path (the minimum free energy path) to perform umbrella sampling simulations [11–13]. Details for the parameter set are listed in Table S3. The free energy distribution of the sampling results along PCV-s is shown in Figure S3.

Table S1 Details of the Targeted MD [4] for T4L-L99A, T4L-L99A,G113A, and. T4L-L99A,G113A,R119P in the Present Work.

Systems	T4L-L99A	T4L-L99A,G113A	T4L-L99A,G113A,R119P
Protein Size	2636 atoms	2639 atoms	2629 atoms
Temperature (K)	300	300	300
Na^+ / Cl^-	10/18	10/18	10/17
Water	10331	10339	10332
Target State	E state	E state	E state
Structural alignment	Cα of α-helices/β-sheets (except for residue 92-140)	Cα of α-helices/β-sheets (except for residue 92-140)	Cα of α-helices/β-sheets (except for residue 92-140)
RMSD Calculation	Heavy atoms of residue 92-140	Heavy atoms of residue 92-140	Heavy atoms of residue 92-140
Force Constant	100, 000 kJ/mol/nm^2	100, 000 kJ/mol/nm^2	100, 000 kJ/mol/nm^2
Frame Record Frequency (ps)	0.2	0.2	0.2
Total Sampling Time (ps)	200 ps	200 ps	200 ps

Table S2. The Parameters Used in TAPS[a] Optimization [5,6] for the Systems in the Present Work.

Systems		T4L-L99A	T4L-L99A,G113A	T4L-L99A,G113A,R119P
Sampling Time Each Iteration		1000 ps	1000 ps	1000 ps
Temperature		300 K	300 K	300 K
Atoms Set	Structural alignment	Cα of α-helices/β-sheets (except 92-140)	Cα of α-helices/β-sheets (except 92-140)	Cα of α-helices/β-sheets (except 92-140)
	RMSD calculation	Heavy atoms of residue 92-140	Heavy atoms of residue 92-140	Heavy atoms of residue 92-140
Tolerant Distance for Neighbor Nodes		0.8 Å	0.8 Å	0.8 Å
Well-tempered Metadynamics [illegible]	Gaussian Height	0.25 kJ/mol	0.25 kJ/mol	0.25 kJ/mol
	Gaussian Width	0.50	0.50	0.50
	Bias Factor	10	10	10
	Length of tMD	30 ps	30 ps	30 ps
Force Constant of tMD		100, 000 kJ/mol/nm^2	100, 000 kJ/mol/nm^2	100, 000 kJ/mol/nm^2

[a]Meaning of parameters in Refs. [5,6].

Table S3 Details of Umbrella Sampling [11] for the Systems in the Present Work.

Systems		T4L-L99A	T4L-L99A,G113A	T4L-L99A,G113A,R119P
Node Number in Final Path		54	52	51
Insert Gap		0.5	0.5	0.5
Atoms Set	Structural alignment	Cα of α-helices/β-sheets (except for residue 92-140)	Cα of α-helices/β-sheets (except for residue 92-140)	Cα of α-helices/β-sheets (except for residue 92-140)
	RMSD calculation	Heavy atoms of residue 92-140	Heavy atoms of residue 92-140	Heavy atoms of residue 92-140
Total Number of Nodes for Umbrella Sampling		108	104	102
PCV-based Umbrella Sampling	Position for z-Wall Potential	0.0064 nm^2	0.0064 nm^2	0.0064 nm^2
	Force Constant for PCV-z	20, 000, 000.0 kJ/mol/nm^2	20, 000, 000.0 kJ/mol/nm^2	20, 000, 000.0 kJ/mol/nm^2
	Force Constant for PCV-s	120 kJ/mol	120 kJ/mol	120 kJ/mol
	Sampling Time	2 ns	2 ns	2 ns

[a]See Figures S3 for the results of umbrella sampling [11].

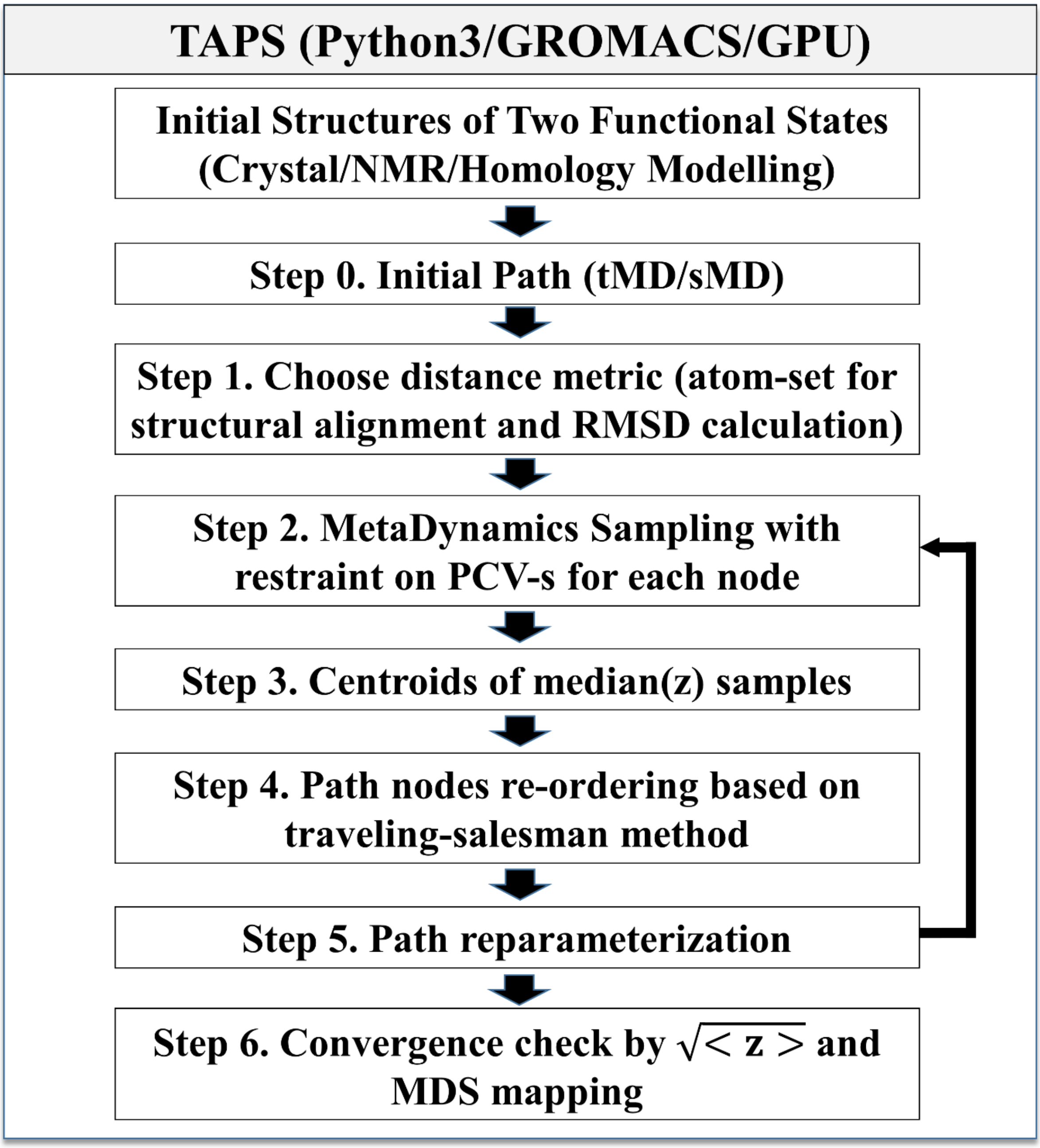

Figure S1. Flowchart of the Travelling-salesman Based Automated Path Searching (TAPS). The initial paths were obtained by targeted MD or steered MD. Well-tempered MetaD simulation for each node is performed parallel on the “progress component” s of the path-collective-variable (PCV) [8,14] with restraint potential on *s*. The backbone nodes of the new path correspond to the centroids of the sampled conformations of each node with the median value of the “deviation component” z of PCV. Re-ordering of these new nodes with a traveling-salesman scheme. Re-parameterization is finally performed to maintain the resolution of the new path (detailed in Ref [5,6]). Convergence is checked by $\sqrt{<z>}$ [8–10] and the visualization of the path nodes on a low-dimensional space obtained by multidimensional scaling (MDS) [7].

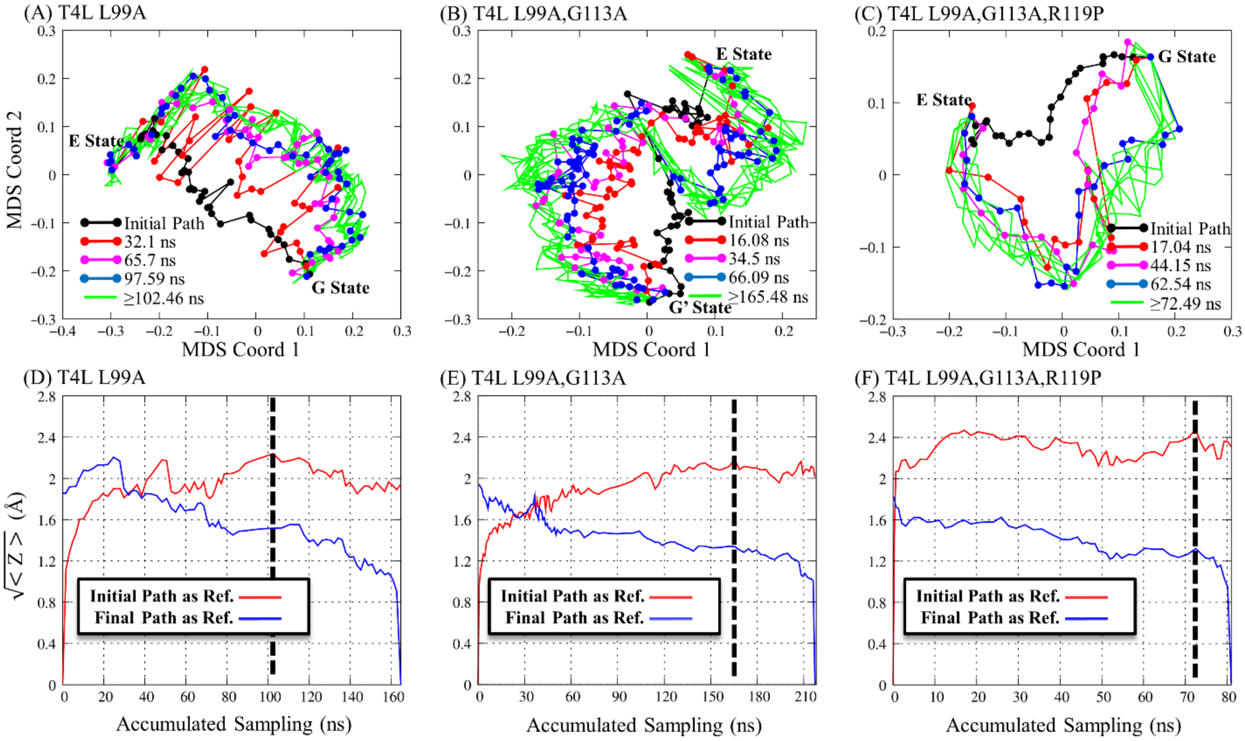

Figure S2. Convergence check. The paths at different TAPS iterations for the T4L-L99A (**A**), the T4L-L99A, G113A (**B**), and the T4L-L99A, G113A, R119P (**C**) are mapped to a two-dimensional space obtained by multidimensional scaling (MDS). The accumulated sampling time for each iteration (perpendicular sampling, additional targeted MD for path reparameterization) is highlighted by a different color. Convergence of the optimization processes for the T4L-L99A (**D**), the T4L-L99A,G113A (**E**), and the T4L-L99A,G113A,R119P (**F**) is measured by the progress of ⟨z⟩ along the accumulated sampling time. Black and orange lines denote two different reference paths respectively: initial path (black) and final path (orange). Convergence time is highlighted by black dashed lines.

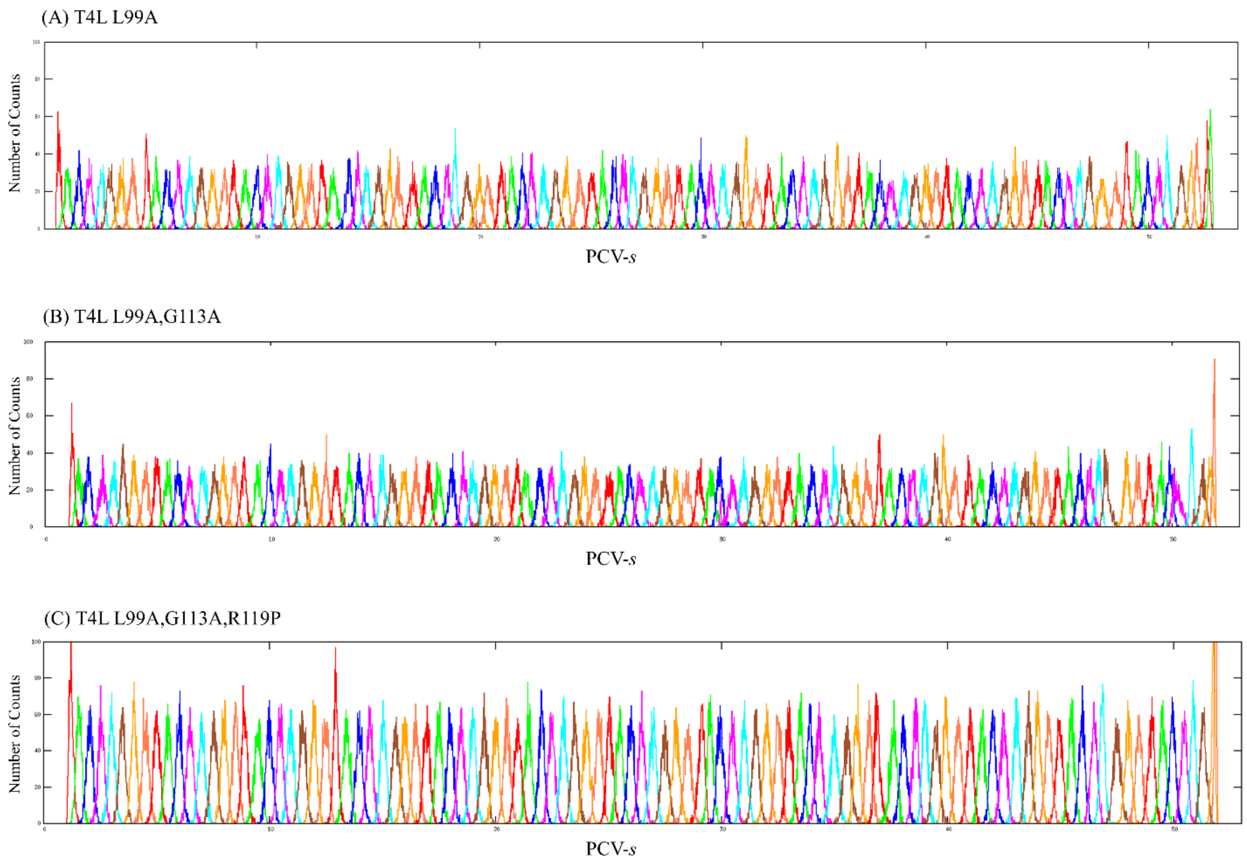

Figure S3. Sample distribution in all windows of the umbrella sampling [7] along the PCV-s for the T4L-L99A (**A**), the T4L-L99A,G113A (**B**), and the T4L-L99A,G113A,R119P (**C**). The initial 200 ps of sampling trajectory were disregarded, and only the last 1.8 ns of sampling was used for WHAM analysis.

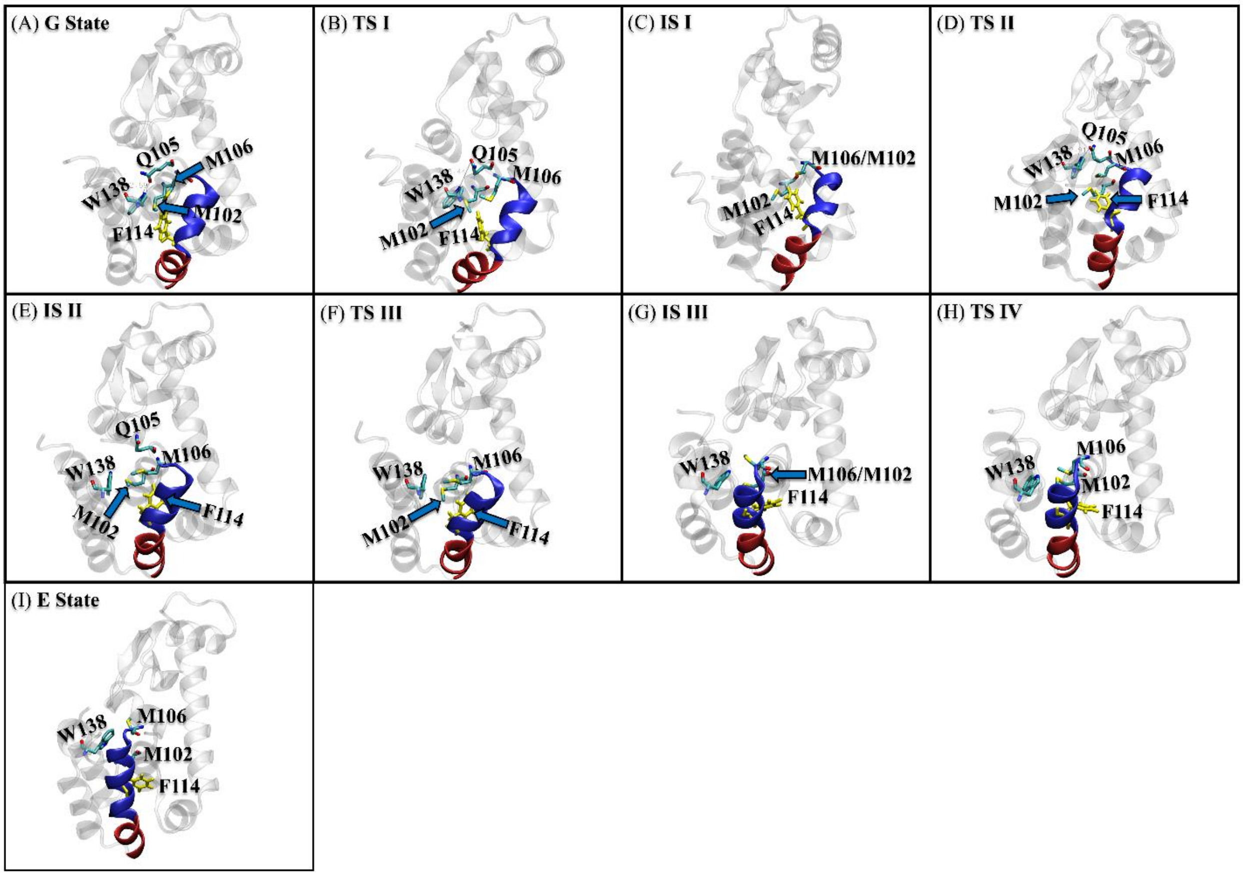

Figure S4. (**A**-I) Complete structures for the transition of the T4L-L99A system from G state to E state. The same ISs and TSs defined in Figure 2A are also shown here. All the structures were displayed by VMD [15].

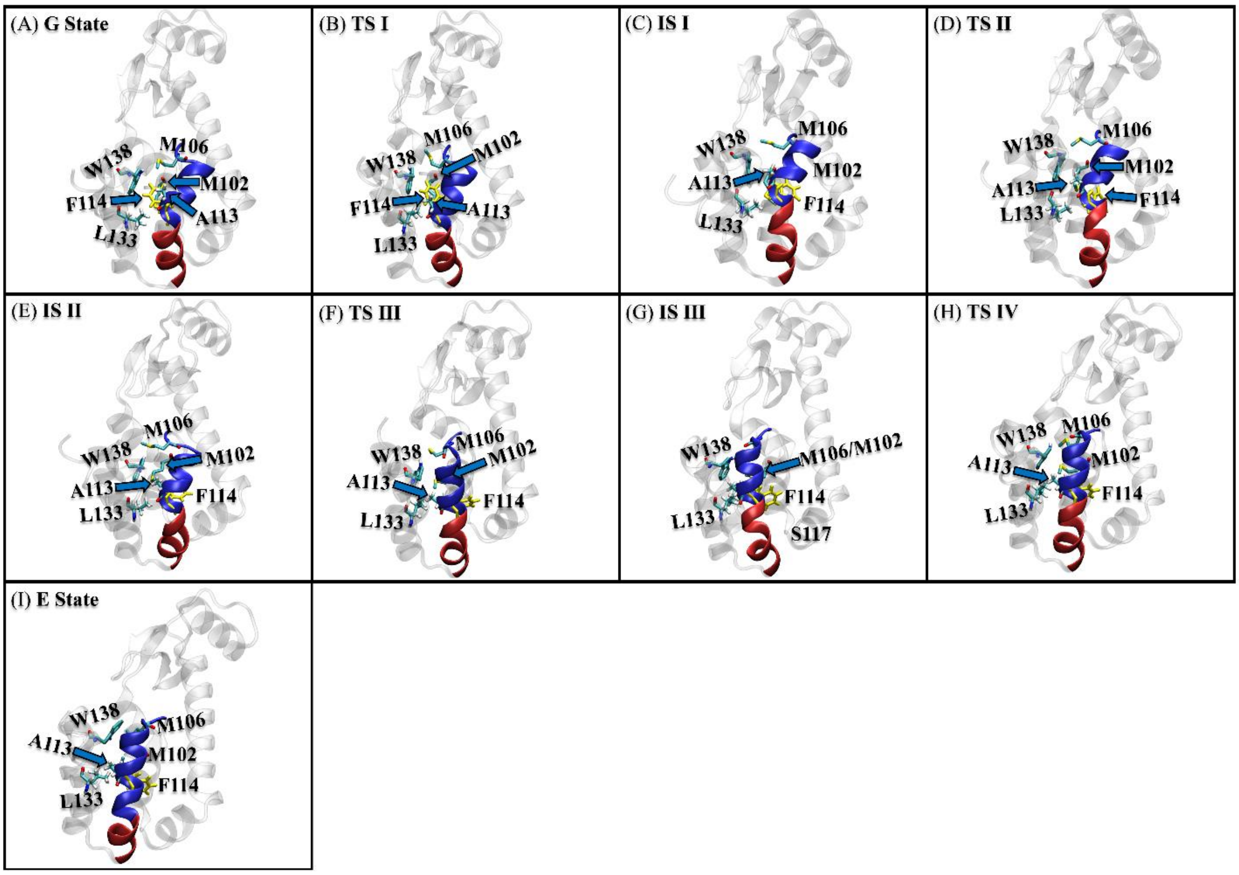

Figure S5. (**A**-**I**) Complete structures for the transition of the T4L-L99A,G113A mutant from G state to E state. The same ISs and TSs defined in Figure 2B are also shown here. All the structures were displayed by VMD [15].

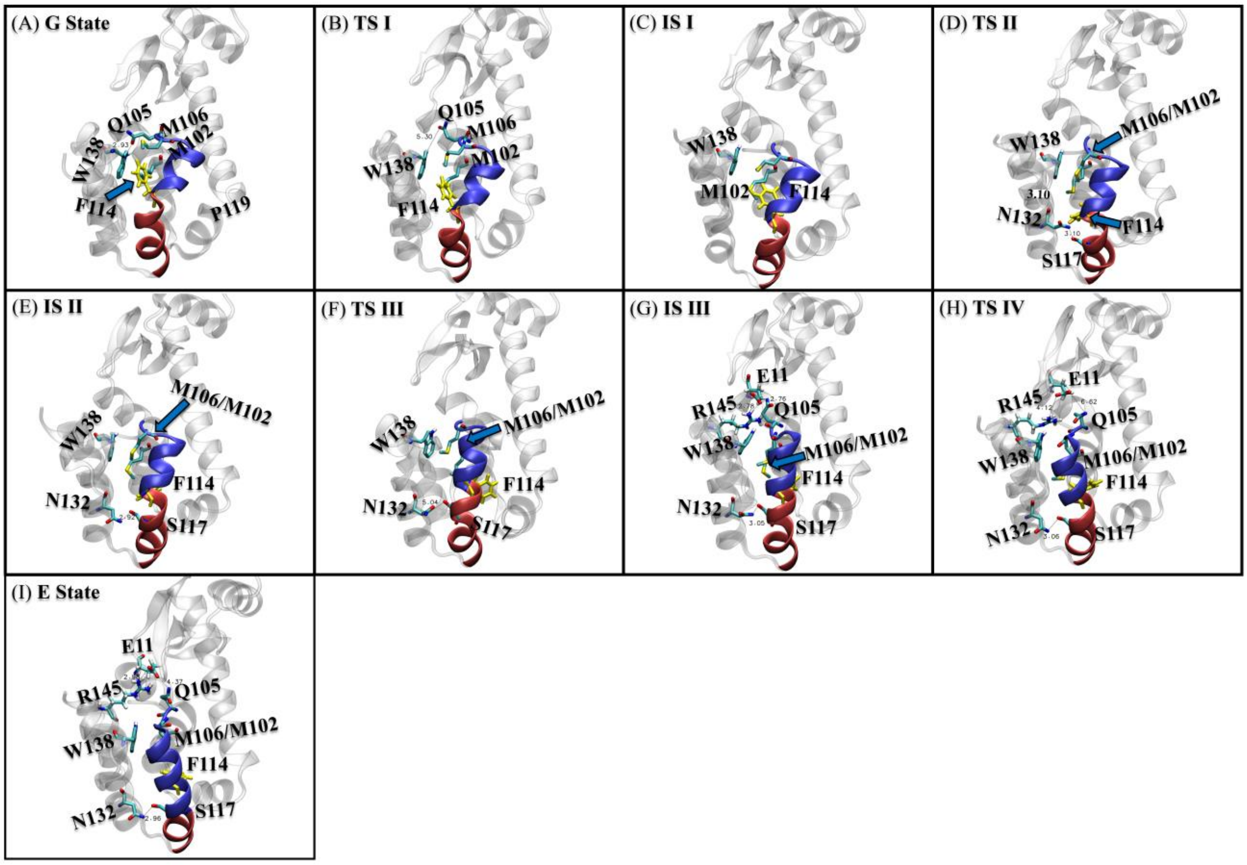

Figure S6. (**A-I**) Complete structures for the transition of the T4L-L99A,G113A,R119P mutant from G state to E state. The same ISs and TSs defined in Figure 2C are also shown here. All the structures were displayed by VMD [15].

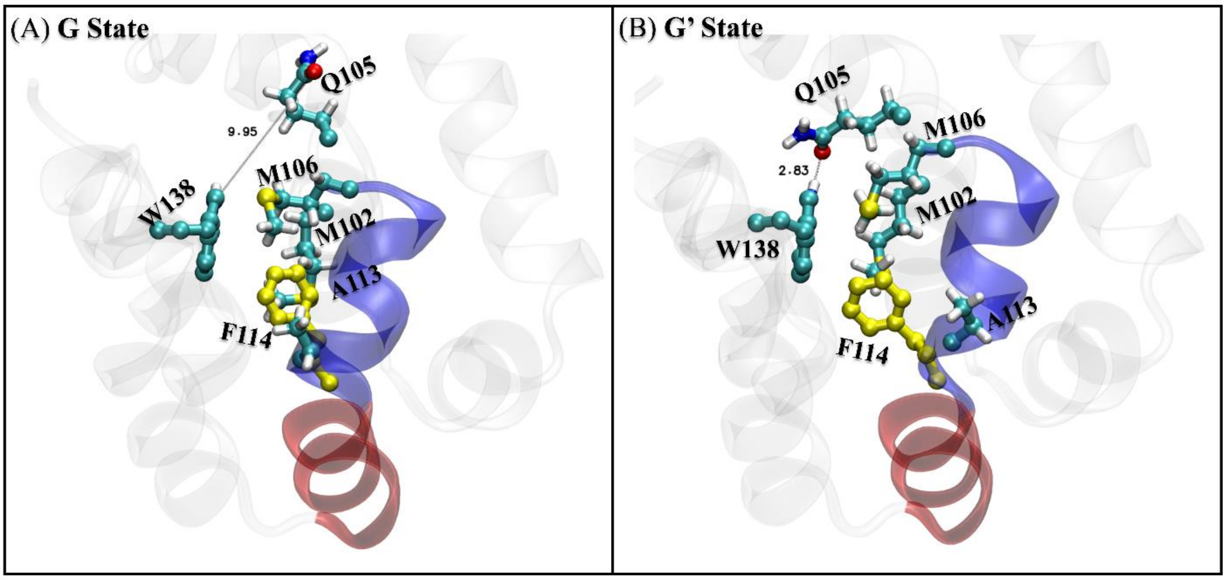

Figure S7. Details of the structures for the G state (**A**) identified in Figure 2B and the initial guess (G′ state, **B**) for the T4L-L99A,G113A mutant. All the structures were displayed by VMD [15].

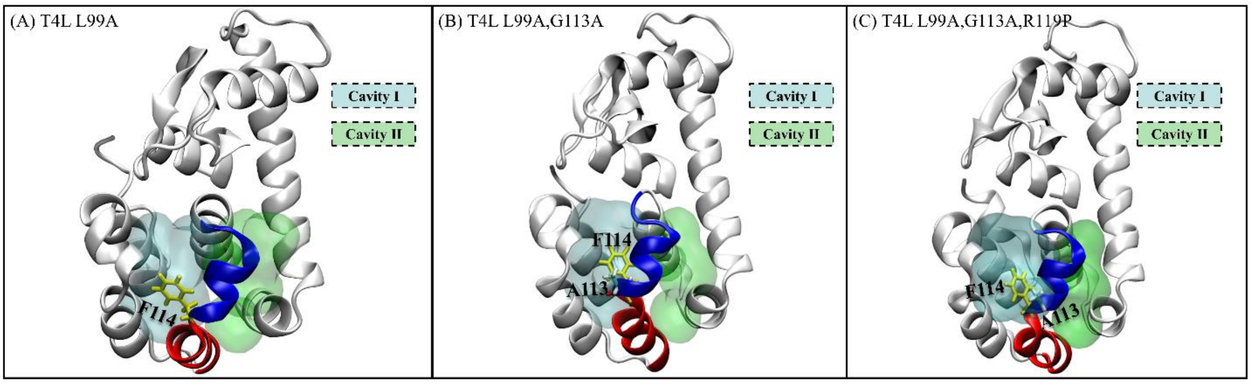

Figure S8. (**A**-**C**) Details of the structures for G state of the three T4L variants: the T4L-L99A (**A**), the T4L-L99A,G113A (**B**), and the T4L-L99A,G113A,R119P (**C**). The two hydrophobic cavities: Cavity I and Cavity II are highlighted by cyan and green colors respectively. All the structures were displayed by VMD [15].

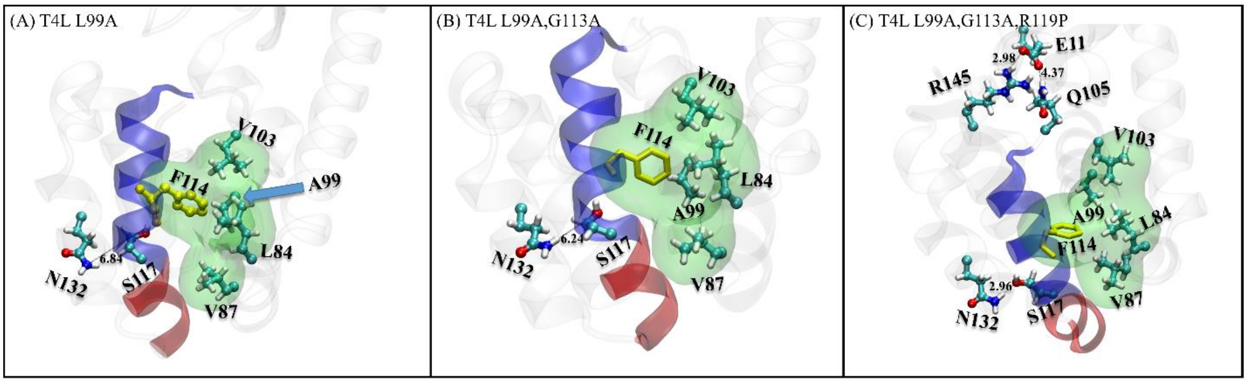

Figure S9. (**A**-**C**) Details of the structures for E state of the three T4L variants: the T4L-L99A (**A**), the T4L-L99A,G113A (**B**), and the T4L-L99A,G113A,R119P (C). The hydrophobic cavity II is highlighted by green color. All the structures were displayed by VMD [15].

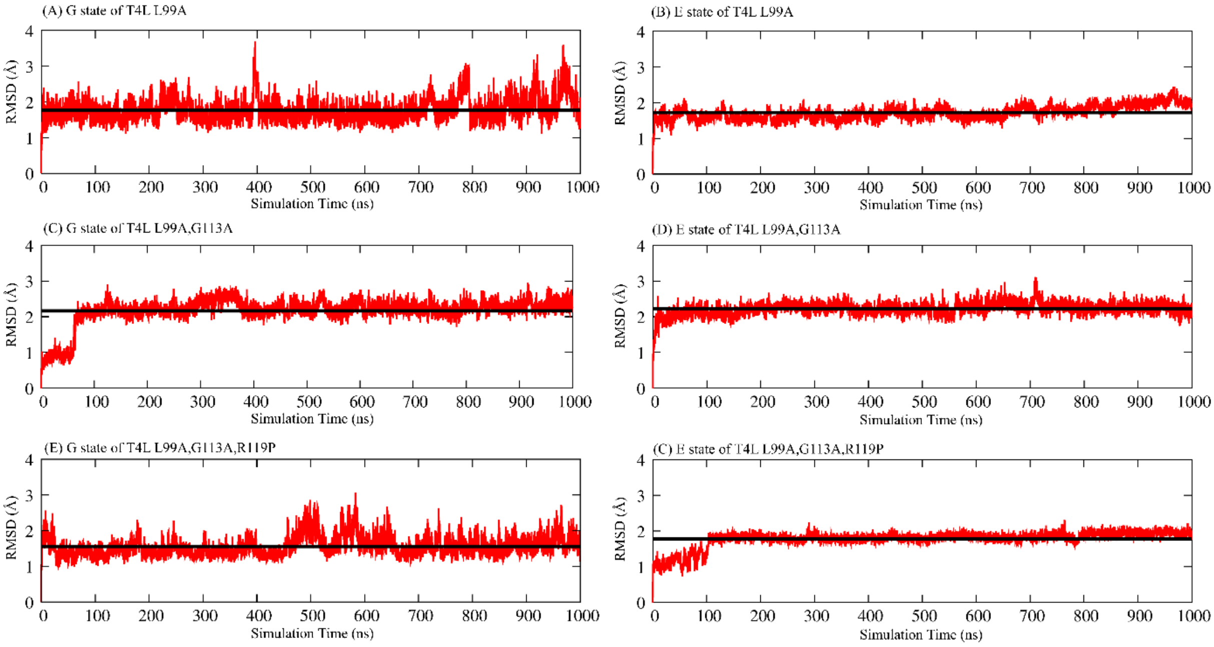

Figure S10. Unbiased MD simulations of the G and E states for all three mutants. Time evolution of the RMSD for the residues (92-140) within Cavity I and Cavity II for (**A**) the G state of T4L-L99A and (**B**) the E state of T4L-L99A; (**C**) the G state of T4L-L99A,G113A and (**D**) the E state of T4L-

L99A,G113A; (**E**) the G state of T4L-L99A,G113A,R119P and (**F**) the E state of T4L-L99A,G113A,R119P.

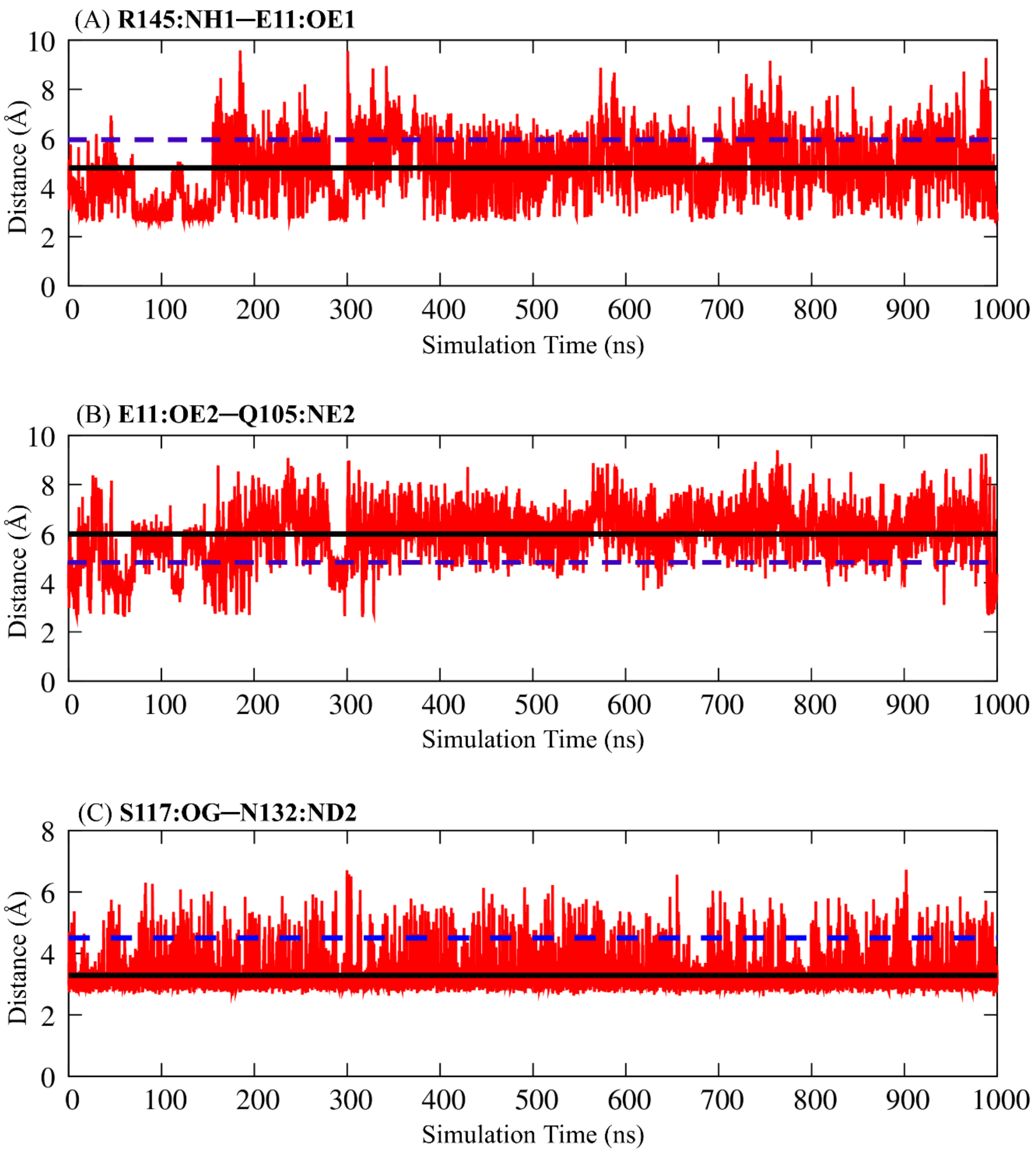

Figure S11. Unbiased MD simulation of the E state of the triple mutant T4L-L99A, G113A, R119P. (**A**) Time evolution of the distance between the atoms R145$_{NH1}$ and E11$_{OE1}$. (**B**) Time evolution of the distance between E11$_{OE2}$ and Q105$_{NE2}$ (cut-off distance 5.0 Å). (**C**) Time evolution of the distance between S117$_{OG}$ and N132$_{ND2}$ (cut-off distance 4.5 Å).

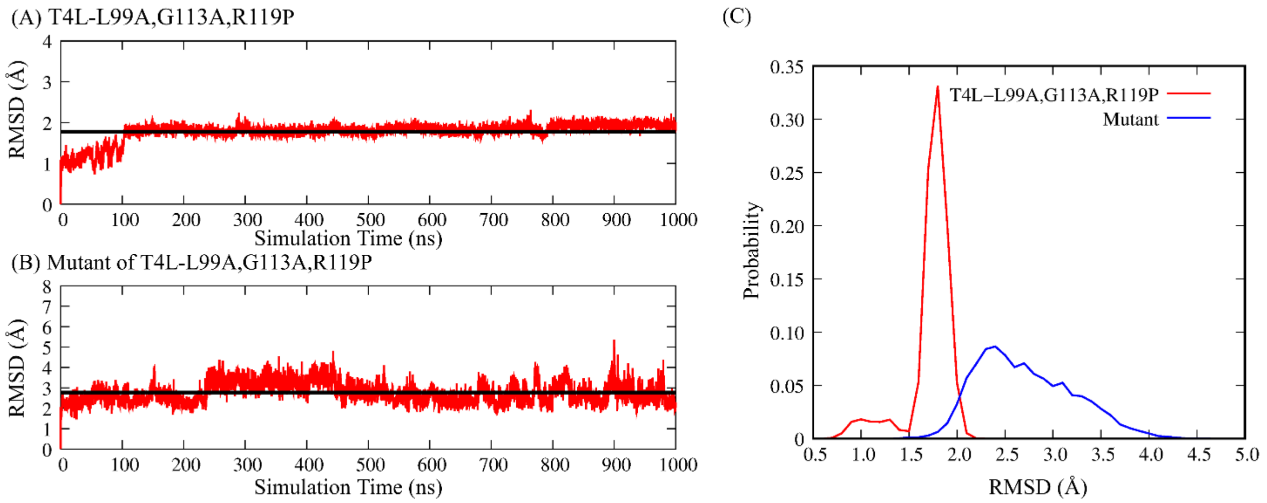

Figure S12. The RMSD for the residues (92-140) within cavity Ⅰ and cavity Ⅱ for triple mutant T4L-L99A, G113A, R119P and a sextuple mutant with three additional mutations Q105L, S117A and R145L. (**A**) Time evolution of RMSD for the E state of T4L-L99A,G113A,R119P; (**B**) Time evolution of RMSD for the E state of the sextuple mutant; (**C**) Probability distribution of the RMSD for the E state of the triple mutant (red line) and sextuple mutant (blue line).

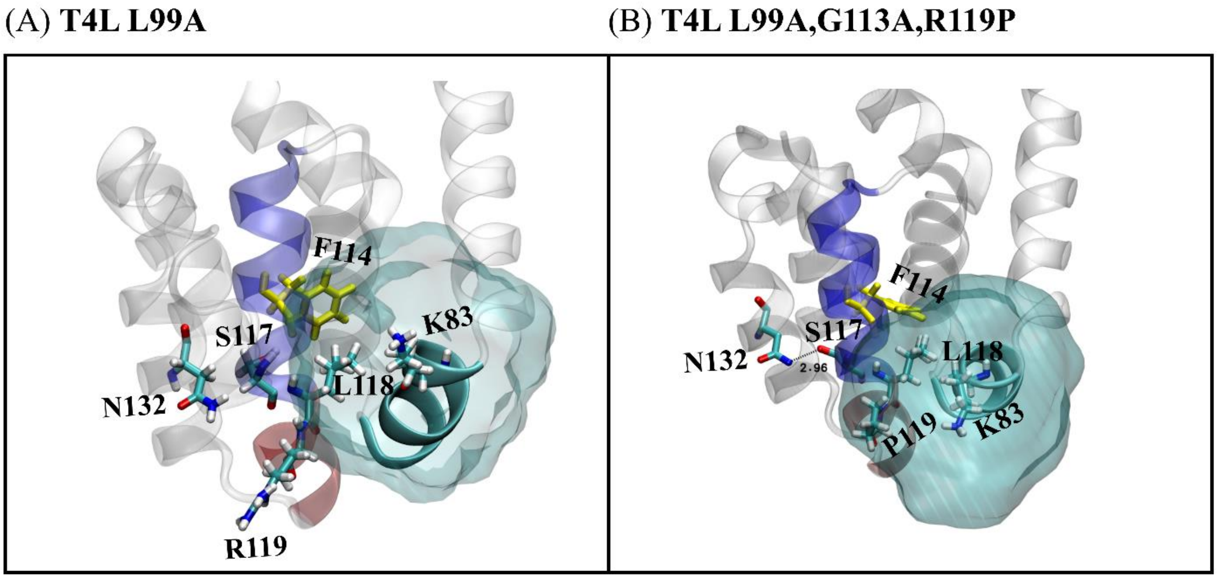

Figure S13. (**A**,**B**) The shape of the space within 6 Å around K83 (transparent, cyan) for the T4L-L99A (**A**), and the T4L-L99A, G113A (**B**). All the structures were displayed by VMD [15].

References

1. Liu, L.; Baase, W.A.; Matthews, B. W. Halogenated benzenes bound within a non-polar cavity in T4 lysozyme provide examples of I⋯S and I⋯Se halogen-bonding. *J. Mol. Biol.*, **2009**, 385, 595-605. https://doi.org/10.1016/j.jmb.2008.10.086.
2. Bouvignies, G.; Vallurupalli, P.; Hansen, D. F.; Correia, B. E.; Lange, O.; Bah, A.; Vernon, R. M.; Dahlquist, F. W.; Baker, D.; Kay, L. E. Solution structure of a minor and transiently formed state of a T4 lysozyme mutant. *Nature* **2011**, *477*, 111-114. https://doi.org/10.1038/nature10349.
3. Eswar, N.; Webb, B.; Marti-Renom, M.A.; Madhusudhan, M.S.; Eramian, D.; Shen M.; Pieper, U.; Sali A. Comparative protein structure modeling using Modeller. *Curr. Prot. Bioinf.*, **2006**, *15*, 1-30. https://doi.org/10.1002/0471250953.bi0506s15.
4. Schlitter, J.; Engels, M.; Krüger, P. Targeted molecular dynamics: a new approach for searching pathways of conformational transitions. *J. Mol. Graphics* **1994**, *12*, 84-89. https://doi.org/10.1016/0263-7855(94)80072-3.
5. Zhu, L. Z.; Sheong, F. K.; Cao, S. Q.; Liu, S.; Unarta, I. C.; Huang, X. TAPS: A traveling-salesman based automated path searching method for functional conformational changes of biological macromolecules. *J. Chem. Phys.* **2019**, *150*, 124105. https://doi.org/10.1063/1.5082633.

6. Xi, K.; Hu, Z.; Wu, Q.; Wei, M.; Qian, R.; Zhu, L. Assessing the Performance of Traveling-salesman based Automated Path Searching (TAPS) on Complex Biomolecular Systems. *J. Chem. Theory Comput.*, **2021**, *17*, 5301-5311. https://doi.org/10.1021/acs.jctc.1c00182.
7. Cox, M.A.A.; Cox, T.F. Multidimensional scaling. *In Handbook of Data Visualization*; Chen, C.-h.; Härde, W.K.; Unwin, A.; *Eds.; Springer: Berlin,* **2008**, pp 315-347. https://doi.org/10.1007/978-3-540-33037-0_14.
8. Laio, A.; Parrinello, M. Escaping free-energy minima. *Proc. Natl. Acad. Sci. USA.* **2002**, *99*, 12562-12566. https://doi.org/10.1073/pnas.202427399.
9. Fox, J.C.; Thomas, M.A.; Dishman, A.F.; Larsen, O.; Nakayama, T.; Yoshie, O.; Rosenkilde, M.M.; Volkman, B.F. Structure-function guided modeling of chemokine-GPCR specificity for the chemokine XCL1 and its receptor XCR1. *Science* **2019**, *12*, eaat4128. https://doi.org/10.1126/scisignal.aat4128.
10. Saunders, M.G.; Voth, G.A. Coarse-graining methods for computational biology. *Annu. Rev. Biophys.*, **2013**, *42*, 73-93. https://doi.org/10.1146/annurev-biophys-083012-130348.
11. Torrie, G.M.; Valleau, J.P. Nonphysical sampling distributions in Monte Carlo free-energy estimation: Umbrella sampling. *J. Comput. Phys.*, **1977**, *23*, 187-199. https://doi.org/10.1016/0021-9991(77)90121-8.
12. Barducci, A.; Bussi, G.; Parrinello, M. Well-tempered metadynamics: a smoothly converging and tunable free-energy method. *Phy. Rev. Lett.* **2008**, *100*, 020603. https://doi.org/10.1103/physrevlett.100.020603.
13. Kumar, S.; Rosenberg, J.M.; Bouzida, D.; Swendsen, R.H.; Kollman, P.A. The weighted histogram analysis method for free-energy calculations on biomolecules. I. The method. *J. Comput. Chem.* **1992**, *13*, 1011-1021. https://doi.org/10.1002/jcc.540130812.
14. Souaille, M.; Roux, B. Extension to the weighted histogram analysis method: combining umbrella sampling with free energy calculations. *Comput. Phys. Commun.* **2001**, *135*, 40-57. https://doi.org/10.1016/S0010-4655(00)00215-0.
15. Humphrey, W.; Dalke, A.; Schulten, K. VMD: visual molecular dynamics. *J. Mol. Graph.* **1996**, *14*, 33-38. https://doi.org/10.1016/0263-7855(96)00018-5.
